# Distribution and Enrichment of Heavy Metals in Fine-Grained Fractions of Crushed Electronic Waste

**DOI:** 10.3390/ma19061222

**Published:** 2026-03-19

**Authors:** Jitka Malcharcziková, Kateřina Skotnicová, Praveen Kumar Kesavan

**Affiliations:** Faculty of Materials Science and Technology, VSB—Technical University of Ostrava, 17. listopadu 2172/15, 708 33 Ostrava, Czech Republic; jitka.malcharczikova@vsb.cz (J.M.); praveenkumar.kesavan@vsb.cz (P.K.K.)

**Keywords:** electronic waste (e-waste), heavy metals, fine-grained fractions, particle size distribution, ED-XRF, SEM–EDS, metal enrichment, dust generation

## Abstract

**Highlights:**

- Heavy metals show strong size-dependent enrichment during e-waste crushing.
- Fine fractions (<0.25 mm) concentrate Pb, Sn, Zn, Cr, Ni, and Cd.
- Lead reaches up to ~2 wt.% in the finest e-waste fraction (<0.15 mm).
- Toxic metals are enriched in fine fractions and processing dust.
- Size fractions enriched in polymers or metals were identified to support efficient recycling.

**Abstract:**

The concentration of heavy metals in the environment has been steadily increasing, raising concerns about their adverse effects on ecosystems and human health. Fine-grained particulate matter is of particular concern due to its enhanced mobility, bioavailability, and potential for inhalation exposure. Facilities involved in the mechanical processing of electronic waste (e-waste) represent a significant potential source of metal-containing fine particles. In this study, crushed e-waste components containing precious metals were separated into particle-size fractions ranging from 3.0 to 0.15 mm using a vibratory sieving system. The elemental composition of the individual fractions was determined by energy-dispersive X-ray fluorescence spectrometry (ED-XRF), while the spatial distribution of selected metals in fine fractions was further investigated using scanning electron microscopy combined with energy-dispersive X-ray spectroscopy (SEM–EDS). The results demonstrate that e-waste contains a wide range of heavy non-ferrous metals whose distribution is strongly dependent on particle size. A pronounced enrichment of metals was observed in the finest fractions, particularly below 0.25 mm. Compared to the coarse fraction (>3 mm), the zinc concentration increased by approximately one order of magnitude, while chromium, nickel, and cadmium exhibited increases of up to approximately 20-fold. Lead showed particularly high enrichment, reaching approximately 2 wt.% in the finest fraction (<0.15 mm), corresponding to nearly fiftyfold enrichment relative to the coarse fraction. Tin concentrations also increased markedly, in some cases by up to two orders of magnitude. Trace amounts of arsenic and selenium were detected in the finest fractions, whereas mercury was not detected. The combined ED-XRF and SEM–EDS results confirm that fine-grained e-waste fractions are the dominant carriers of hazardous metals and respirable particles generated during mechanical processing. These findings highlight the dual character of fine fractions as both a critical environmental and occupational risk and a potentially valuable secondary resource. The study emphasizes the importance of controlled handling, effective dust management, and targeted processing strategies to minimize human exposure while enabling efficient recovery of valuable metals from e-waste.

## 1. Introduction

The concentration of heavy metals in the environment has been steadily increasing, raising serious concerns regarding their adverse effects on ecosystems and human health. Heavy metals are persistent, non-biodegradable, and capable of accumulating in environmental compartments, where they may exert carcinogenic, mutagenic, teratogenic, and neurotoxic effects even at low concentrations. Among the most hazardous elements are Hg and Pb, which are well-established neurodevelopmental toxicants and pose significant risks to vulnerable population groups [1,2,3].

A substantial contribution to environmental contamination by heavy metals arises from the production, use, and end-of-life processing of electrical and electronic equipment. Waste electrical and electronic equipment (WEEE), commonly referred to as electronic waste (e-waste), represents one of the fastest-growing waste streams worldwide due to rapid technological development, shortened product lifecycles, and increasing consumption [4,5,6,7]. Recent studies indicate that global e-waste generation exceeds 60 million tonnes annually, while only a limited fraction is formally collected and recycled, resulting in considerable environmental, health, and societal risks [4,5]. E-waste is a highly heterogeneous material stream composed of metals, polymers, glass, and ceramics, often containing hazardous substances such as Pb, Cd, Hg, Cr, Ni, Cu, and Zn [3,8,9].

Improper handling and recycling of e-waste, particularly during dismantling, shredding, and mechanical processing, leads to the release and redistribution of hazardous substances into different waste fractions. These processes play a key role in the mobilization of metals and their subsequent partitioning within the processed material [7,10]. Of particular concern is the generation of fine-grained particulate matter during mechanical treatment, as fine particles exhibit enhanced metal enrichment, increased surface reactivity, and greater environmental mobility compared to bulk material [1,3].

In industrial practice, waste electrical and electronic equipment (WEEE) is typically processed into several particle-size fractions that are subsequently treated separately according to their material composition and physical characteristics [11,12]. Mechanical comminution represents a key step in the preparation of e-waste for further processing. Various crushing and milling techniques are applied, including hammer milling, shredding, and cryogenic milling, each influencing the particle size distribution and the proportion of fine and dust fractions generated during processing [13,14,15].

Coarser fractions produced during mechanical treatment are often dominated by polymeric materials and can be separated and utilized as sorted or mixed plastic fractions [11,12]. In contrast, metals are predominantly concentrated in intermediate and fine particle fractions [16]. Larger metallic pieces can be efficiently separated using magnetic and eddy current separation systems, enabling the recovery of ferrous metals and non-ferrous metals such as copper and aluminium with relatively high purity [17].

However, a significant portion of metals remains associated with fine and dust fractions generated during mechanical processing of WEEE. These fine fractions may contain considerable amounts of valuable metals such as Cu, Au, Ag, Pb, Zn, and Sn, but their recovery is technologically more challenging [15]. Various advanced separation and recovery approaches have therefore been developed, including physical separation techniques based on differences in magnetic properties, electrical conductivity, or density [15,17].

In addition, metallurgical approaches are widely applied for metal recovery from fine WEEE fractions. These include pyrometallurgical, hydrometallurgical, and increasingly also biological processes, often applied in combination to improve metal recovery efficiency [15,17]. Thermal treatment technologies such as pyrolysis or gasification have also been investigated as promising approaches for concentrating metals and reducing the proportion of non-metallic components. These processes can produce metal-rich residues suitable for further metallurgical processing [18,19]. Other approaches include oxidative refining or smelting processes in which selected metals such as Cu, Fe, or Pb act as collectors for valuable metals including precious metals such as Au or Pd [20,21,22].

Fine metal-bearing residues can also originate from related waste streams such as fly ash generated during thermal treatment of electronic waste or waste incineration with energy recovery, where metal concentrations depend strongly on the applied technology and operating conditions [23].

Fine-grained fractions of e-waste, typically classified as particulate matter PM10 and PM2.5, represent a critical exposure pathway for both workers and surrounding communities. These particles are characterized by a high specific surface area, which promotes the preferential adsorption and accumulation of heavy non-ferrous metals, thereby increasing their bioavailability and toxic potential [1,2,3,24,25]. Several studies have demonstrated that concentrations of metals such as Pb, Cd, Hg, Cu, Ni, and Zn are significantly higher in finer particle size fractions, confirming that mechanical comminution leads to preferential enrichment of heavy non-ferrous metals in PM10 and PM2.5 fractions [1,2,3,24,25].

From a toxicological perspective, PM10 and especially PM2.5 particles pose a severe health risk due to their ability to penetrate deep into the respiratory system. While PM10 particles are predominantly deposited in the upper and central airways, PM2.5 particles can reach the alveolar region of the lungs, where particle-bound metals may enter systemic circulation [1,2]. Occupational exposure studies have reported elevated concentrations of heavy metals in airborne dust and biological samples of workers involved in e-waste recycling activities, indicating insufficient containment and protection measures during processing operations [1,2].

Recent studies further confirm the potential health implications associated with exposure to metal-bearing dust generated during e-waste recycling. Measurements performed in recycling facilities have detected elevated concentrations of heavy metals in airborne particulate matter, including Pb, Cd, Cr, and Hg, particularly in fine particle fractions [24]. Biomonitoring studies have also reported increased levels of metals in biological samples such as blood and urine of workers exposed to airborne particulate matter during recycling operations [25]. Large-scale occupational exposure assessments conducted within the HBM4EU project have demonstrated measurable internal exposure of recycling workers to metals including chromium, cadmium, mercury, and lead during e-waste processing activities [1]. In addition, studies carried out in informal recycling communities have reported significant health risks associated with chronic exposure to metal-contaminated dust generated during dismantling and mechanical processing of e-waste [3].

In addition to the global dimension of electronic waste, the Czech Republic represents an example of a country with a well-established WEEE collection and take-back system. Despite high collection rates and compliance with European legislative targets, increasing volumes of formally collected e-waste place growing demands on downstream treatment and recycling operations. Mechanical processing of WEEE inevitably generates fine-grained particulate fractions, which may become enriched in heavy non-ferrous metals. Consequently, even in countries with advanced e-waste management systems, fine-grained fractions remain a relevant environmental and occupational concern [26].

Fine particulate matter generated during e-waste recycling can remain airborne for extended periods and be transported over long distances, contributing to regional dispersion of metal contaminants [3,8]. Although regulatory frameworks such as the European Restriction of Hazardous Substances (RoHS) Directive aim to limit the use of certain toxic metals in new electrical and electronic equipment, legacy products and long device lifetimes ensure that hazardous elements remain present in current e-waste streams [7,9].

Given the complexity and evolving composition of electronic waste, systematic monitoring of metal distribution across different waste fractions is essential. While previous studies have often focused on total metal concentrations or bulk material composition, less attention has been paid to the size-dependent distribution of metals within defined particle-size fractions generated during mechanical processing. In particular, the role of fine particles as carriers of hazardous metals and respirable dust remains insufficiently characterized. Therefore, this study provides a detailed size-resolved evaluation of heavy metal distribution in crushed e-waste fractions, combining sieve classification, particle size distribution analysis, ED-XRF chemical characterization, and SEM–EDS elemental mapping. By targeting these fractions, the study aims to improve understanding of metal partitioning behaviour, potential environmental dispersion, and associated health risks linked to inhalable particulate matter.

## 2. Materials and Methods

### 2.1. Preparation of Experimental Material

**Figure 1:**
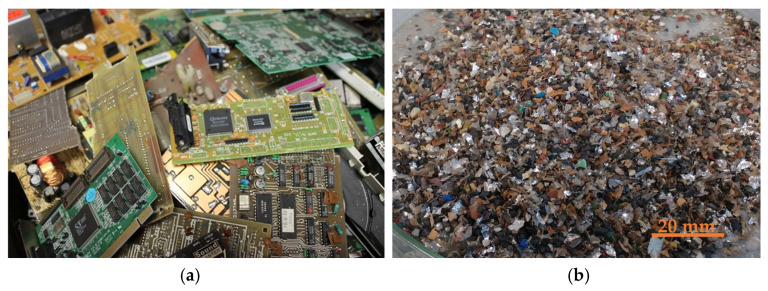
Experimental e-waste material used in this study: (**a**) separated e-waste parts; (**b**) shredded material after mechanical size reduction.

Separated parts of e-waste containing a higher proportion of precious metals were used as the experimental material. The input material is shown in Figure 1a. Prior to further processing, the e-waste was mechanically crushed using a shearing knife mill SM 2000 (Retsch GmbH, Haan, Germany). The resulting shredded material (Figure 1b) was subsequently used for all further experimental procedures.

After crushing, the material was subjected to density separation in water in order to distinguish light and heavy fractions. The light fraction represented approximately 13 % of the total mass, while the heavy fraction accounted for about 87 %. The heavy fraction was subsequently used for further processing.

### 2.2. Classification into Size Fractions

The crushed e-waste material was classified into size fractions by sieve analysis. The separation was performed using a vibrating sieve shaker, in which the crushed e-waste was divided into size fractions ranging from 3.0 to 0.15 mm. A vibrating sieve shaker S49 (Xinxiang Gaofu Machinery Co., Xinxiang, China) was used for the separation. The material was classified into six size fractions using five screens installed in the sieving device. Efficient separation was achieved through intensive vibration combined with the use of silicone spacer rings and balls, which effectively prevented sieve clogging and ensured stable separation conditions. The sample (total mass 2 kg) was subjected to the sieving procedure under fixed operating conditions. After sieving, the mass of each size fraction was determined, and the individual fractions were subsequently subjected to chemical analysis to determine the contents of selected elements.

### 2.3. Chemical Composition Analysis

From each obtained size fraction, representative samples weighing approximately 50 g were collected for chemical composition analysis. Samples of the crushed e-waste as well as the individual size fractions were placed into dedicated sample cups covered with a thin X-ray-transparent film suitable for X-ray fluorescence measurements. The chemical composition was determined by energy-dispersive X-ray fluorescence spectroscopy (ED-XRF) using a Delta Professional spectrometer (Olympus Corporation, Tokyo, Japan), equipped with a dedicated holder for the analysis of small and loose samples. The obtained results are of an informative and semi-quantitative character; however, they provide sufficient descriptive and comparative capability for evaluating the distribution of selected elements among the individual size fractions.

### 2.4. Microscopic Observation and Elemental Mapping

Optical microscopy (OM) and scanning electron microscopy (SEM) were employed for morphological observation and elemental characterization of the crushed e-waste and selected size fractions. A stereomicroscope Olympus SZX12 (Olympus Corporation, Japan) was used for visual documentation of the morphology and heterogeneity of coarser size fractions obtained after size classification. Scanning electron microscopy was performed using a Quanta 450 FEG scanning electron microscope (Thermo Fisher Scientific, Waltham, MA, USA) equipped with an energy-dispersive X-ray spectrometer (EDS). SEM–EDS was applied mainly to fine e-waste fractions in order to qualitatively assess elemental distribution. Elemental mapping was used to identify and visualize the spatial distribution of selected heavy and potentially toxic non-ferrous metals.

### 2.5. Particle Size Distribution Analysis

The particle size distribution (PSD) of the fine e-waste fractions below 0.15 mm, together with the collected fine dust, was determined using a laser diffraction particle size analyser, Mastersizer 3000 (Malvern Instruments Ltd., Malvern, UK), equipped with a wet dispersion unit. Prior to measurement, the samples were carefully homogenized to ensure representative results. The PSD curves were obtained based on laser diffraction data and evaluated using the Mastersizer 3000 v3.2 software. Characteristic particle size parameters D10, D50, and D90 were determined to describe the particle size distribution of the fine e-waste fractions.

## 3. Results

### 3.1. Determination of the Proportion of Size Fractions

**Figure 2:**
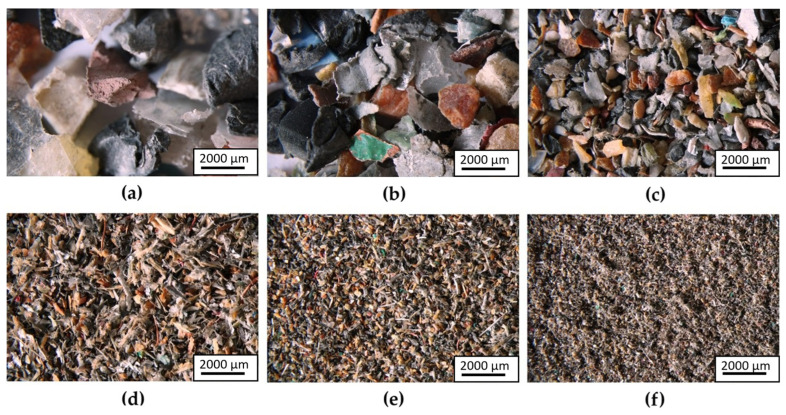
Size fractions obtained after sieving: (**a**) >3 mm; (**b**) 3–1 mm; (**c**) 1–0.5 mm; (**d**) 0.5–0.25 mm; (**e**) 0.25–0.15 mm; (**f**) <0.15 mm.

Crushed e-waste parts containing selected metals were separated into size fractions ranging from 3.0 to 0.15 mm using a vibrating sieve shaker. A total of six size fractions were obtained and subsequently analysed. Representative optical micrographs of the individual size fractions obtained after sieving are shown in Figure 2a–f. The images illustrate a progressive change in particle morphology and degree of fragmentation with decreasing particle size. The fractions shown in Figure 2a,b are dominated by relatively large, angular and irregular fragments with clearly distinguishable individual particles and pronounced material heterogeneity. In the intermediate fractions (Figure 2c,d), the particles appear more fragmented, with an increasing proportion of thin, elongated and plate-like fragments and a more complex surface morphology. The fractions shown in Figure 2e,f are dominated by fine particles with predominantly irregular morphology. Compared to the coarser fractions, individual particles are less distinctly resolved. This observation is consistent with a higher degree of mechanical fragmentation induced by shear milling and subsequent sieving.

**Figure 3:**
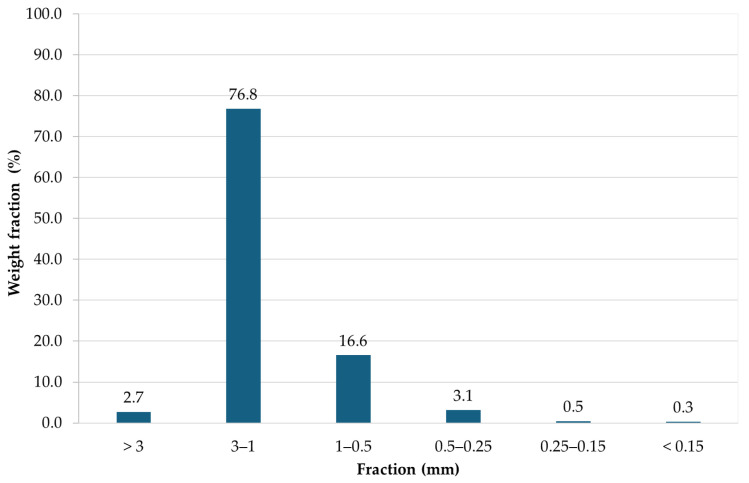
Weight fraction distribution of size fractions after vibratory sieving.

All separated fractions were weighed, and their respective weight ratios were calculated. The fractions were labeled from 1 to 6, ordered from the coarsest (fraction 1) to the finest (fraction 6), with the following size ranges: fraction 1 (>3 mm), fraction 2 (3–1 mm), fraction 3 (1–0.5 mm), fraction 4 (0.5–0.25 mm), fraction 5 (0.25–0.15 mm), and fraction 6 (<0.15 mm). The overall weight fraction distribution of the size fractions is illustrated in Figure 3.

The dominant portion of the material was concentrated in the 3–1 mm fraction, which accounted for approximately 77% of the total mass (Figure 3). Together with the 1–0.5 mm fraction, nearly 90% of the total mass was distributed within the particle size range of 3–0.5 mm. The percentages shown in Figure 3 represent the weight fraction of each particle size class relative to the total mass of the sieved sample. The finer fractions below 0.5 mm represented only a minor proportion of the total mass. However, despite their low weight contribution, these fine fractions are of particular interest due to the elevated contents of heavy and potentially toxic metals, as discussed in the following sections.

In addition to the size fractions obtained by sieving, fine dust generated during the crushing, handling, and separation of the e-waste material was collected and analysed separately. Although this fine dust does not represent a defined sieve fraction, it constitutes an important material stream due to its very small particle size and potential relevance from environmental and occupational health perspectives. The results of its particle size distribution and elemental composition are presented in the following sections.

### 3.2. Particle Size Distribution

**Figure 4:**
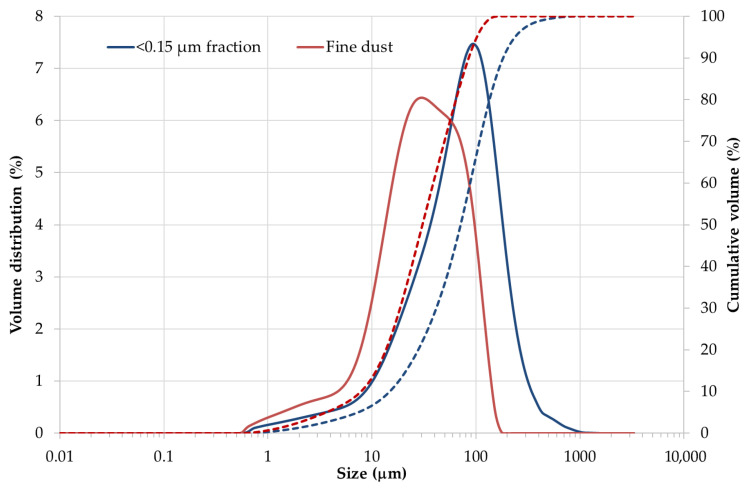
Particle size distribution (PSD) curves of fine e-waste materials determined by laser diffraction: fraction < 0.15 mm and collected fine dust. Solid lines represent differential volume distributions, while dotted lines represent cumulative volume distributions.

Special attention was paid to the very fine fraction of crushed e-waste with particle sizes below 0.15 mm, as well as to the fine dust fraction collected during material processing and sorting stages. Particle size distribution (PSD) analysis was performed for both materials using laser diffraction, and the resulting differential and cumulative distribution curves are presented in Figure 4. The differential curves represent the volume-based particle size distribution, while the cumulative curves show the cumulative volume fraction of particles smaller than a given particle size.

As shown in Figure 4, the separated <0.15 mm (fraction 6) e-waste fraction is characterized by a relatively narrow particle size distribution with a median particle size *D_v_*(50) of 71.4 µm. The lower and upper characteristic particle sizes were determined as *D_v_*(10) = 14.8 µm and *D_v_*(90) = 184 µm, respectively, indicating that 80% of the particles fall within this size interval. It should be noted that although the material was classified using a 150 µm sieve, the particle size distribution analysis revealed the presence of particles with equivalent diameters exceeding this nominal size. This effect can be attributed to particle shape irregularity, agglomeration of fine particles, and the fundamental differences between sieve-based classification and laser diffraction measurement, which determines an equivalent spherical volume diameter.

In contrast, the fine dust fraction shown in Figure 4 exhibits a broader particle size distribution shifted toward smaller particle sizes. The median particle size of the dust fraction was *D_v_*(50) = 30.7 µm, while *D_v_*(10) = 7.84 µm and *D_v_*(90) = 85.5 µm, confirming a substantially higher contribution of fine particles compared to the <0.15 mm fraction.

Evaluation of the cumulative particle size distribution curve presented in Figure 4 enabled quantitative determination of respirable particle fractions. For the <0.15 mm e-waste fraction, approximately 6–7 vol.% of particles were smaller than 10 µm (PM10) and about 2 vol.% were smaller than 2.5 µm (PM2.5). In comparison, the fine dust fraction contained a markedly higher proportion of respirable particles, with approximately 14–15 vol.% PM10 and about 3.5 vol.% PM2.5.

### 3.3. Content of Selected Elements in Individual Size Fractions and Their Spatial Distribution

The contents of metals in the individual size fractions were determined using energy-dispersive X-ray fluorescence spectroscopy (ED-XRF). Average concentrations obtained from repeated measurements are summarized in Table 1 and reported in ppm and wt.%.

**Figure 5:**
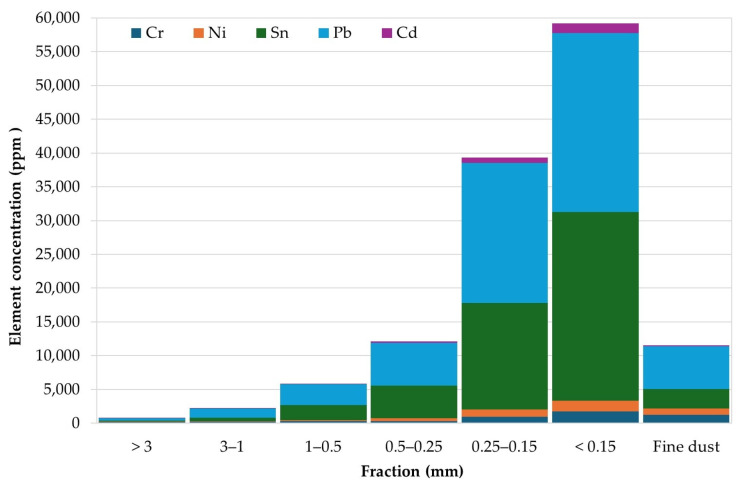
Content of selected metals (Cr, Ni, Sn, Pb, Cd) in individual size fractions of the crushed e-waste sample, as determined by energy-dispersive X-ray fluorescence (ED-XRF) analysis.

A clear relationship between metal content and particle size fraction was observed. Increasing metal contents with decreasing particle size indicate a preferential enrichment of metals in finer fractions. Figure 5 illustrates the contents of selected metals, including Cr, Ni, Sn, Pb, and Cd, in the individual size fractions.

A pronounced increase in metal contents was observed in fractions smaller than 0.25 mm (fractions 5 and 6). Zn content increased by approximately one order of magnitude in the finest fraction compared to the coarsest fraction. Cr, Ni, and Cd exhibited increases of up to approximately twentyfold. In contrast, Pb reached concentrations of approximately 2 wt.% in fractions below 0.25 mm, corresponding to several ten-fold (up to ~70-fold) enrichment relative to coarse fractions. Sn showed substantial enrichment, in some cases up to two orders of magnitude. Hg was not detected.

Besides Pb and Cd, elevated contents of Ni, Cr, Sb, and Tl were detected predominantly in fine fractions. As and Se were also identified. The As concentration increased from approximately 349 ppm in fraction 5 to 636 ppm in fraction 6, while selenium concentrations ranged from approximately 100 to 150 ppm.

The presence of precious metals, particularly Ag and Au, was confirmed in all size fractions. Ag showed a clear tendency toward enrichment in finer fractions, especially below 0.5 mm, where its content reached values of several thousand ppm. This behaviour can be attributed to the occurrence of Ag in conductive tracks, contacts, and surface coatings, which are prone to fragmentation during mechanical size reduction. Au was detected at lower concentrations, typically on the order of several hundred ppm; however, noticeable enrichment was also observed in the fine fractions, particularly below 0.25 mm. This trend reflects the association of Au with thin surface layers, contact pads, and bonding wires, which are preferentially liberated during crushing and subsequently accumulate in fine particle classes.

In contrast, Bachér et al. [16] reported the highest concentrations of Ag and Au in slightly coarser dust fractions (0.5–1.0 mm), which was attributed to incomplete liberation of noble metals that remain attached to fragments of PCB components. The differences between studies may therefore reflect variations in feed composition and the degree of liberation during mechanical processing.

The fine dust generated during processing also contains a significant proportion of hazardous metals, as shown in Table 1. It contains significant amounts of chromium, nickel, tin and lead. Considering the size of dust particles with a significant proportion of PM10 and a non-negligible proportion of PM2.5 (Figure 4), this component poses a major health and environmental risk. However, from a health and occupational safety perspective, this fraction represents a particularly critical component. Due to its small particle size, fine dust is readily inhalable and capable of penetrating deep into the respiratory system, including the alveolar region. Even relatively low concentrations of toxic metals may therefore pose significant health risks, as inhalation exposure is strongly influenced by particle size, surface area, and metal bioavailability rather than by mass concentration alone. Moreover, metals associated with fine dust particles are often present in surface-bound or weakly bonded forms, which may enhance their dissolution in lung fluids and increase their toxicological relevance. Similar elevated concentrations of metals in fine fractions generated during crushing of mobile phones and printed circuit boards have also been reported in previous studies [13,16], confirming that fine particulate fractions may represent an important carrier of both hazardous and valuable metals.

The enrichment of precious metals in fine fractions highlights the dual significance of these size classes. On one hand, they represent the most critical component in terms of environmental and occupational risks due to elevated contents of hazardous metals; on the other hand, they constitute a potentially valuable secondary resource for the recovery of precious metals. These findings underline the importance of selective processing strategies that combine effective dust control with targeted recovery of valuable metals from fine e-waste fractions.

**Figure 6:**
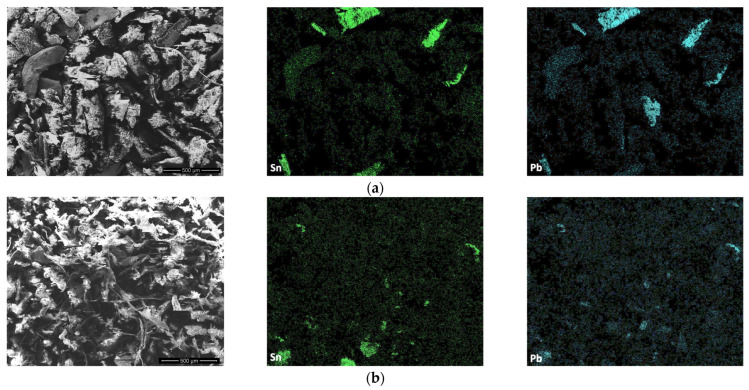
SEM–EDS elemental maps showing the spatial distribution of Pb and Sn in (**a**) fraction 5 (0.25–0.15 mm) and (**b**) fraction 6 (<0.15 mm).

The spatial distribution of selected elements was further investigated using scanning electron microscopy combined with energy-dispersive X-ray spectroscopy (SEM–EDS). SEM/SE micrographs and corresponding elemental maps of Pb and Sn for fractions 5 (0.25–0.15 mm) and 6 (<0.15 mm) are presented in Figure 6. The SEM–EDS results show that Pb and Sn are distributed across the analysed areas, indicating that these elements are not confined to isolated particles but are widely dispersed within the material.

In fraction 5 (0.25–0.15 mm), Pb- and Sn-rich regions are predominantly associated with individual particles and localized domains that can be clearly distinguished within the microstructure. In fraction 6 (<0.15 mm), Pb and Sn occur mainly as numerous fine-scale features dispersed across the entire analysed area. The elemental maps reveal a higher degree of spatial dispersion, with metal-bearing particles present at micrometre and sub-10 µm length scales and only limited occurrence of larger, well-defined enriched regions. The presence of Pb- and Sn-containing particles at such small size scales is of particular concern, as particles of this size can be easily resuspended during mechanical processing and separation of e-waste, thereby increasing the potential for inhalation exposure.

**Figure 7:**
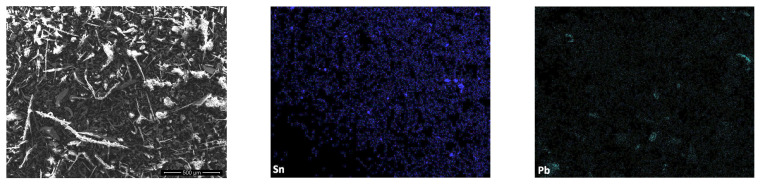
SEM–EDS elemental maps showing the spatial distribution of Pb and Sn in fine dust.

Figure 7 presents the SEM micrograph and corresponding EDS elemental maps of Pb and Sn in the collected fine dust. In comparison with fraction 6 (<0.15 mm), the fine dust exhibits an even finer-scale and more continuous spatial distribution of both elements. Pb and Sn occur predominantly as numerous micron- and submicron-sized features distributed across the entire analysed area, with only a limited presence of larger, well-defined metal-rich regions. Relative to fraction 6, where Pb- and Sn-bearing particles can still be locally associated with discrete fine particles, the fine dust is characterized by a higher degree of dispersion and a more uniform elemental distribution. This indicates that the fine dust represents a more advanced stage of particle fragmentation and metal liberation. The pronounced dispersion of Pb and Sn in the fine dust suggests that this material acts as an efficient carrier of hazardous metals. As a consequence, the fine dust poses an increased potential for particle mobilization and inhalation during handling and processing of e-waste, thereby further amplifying the associated environmental and occupational health risks.

The occurrence of Pb is consistent with older types of electronic waste containing lead-based solders, which are still present in current recycling streams. Fine particles enriched in Pb, Sn, and other hazardous metals may therefore act as carriers of toxic substances and contribute to their long-range transport in the environment. These observations are in good agreement with the ED-XRF results, which demonstrated pronounced enrichment of lead and tin in the fine size fractions.

Overall, the combined results of ED-XRF chemical analysis and SEM–EDS elemental mapping confirm that the finest fractions of crushed e-waste represent the most critical component from both environmental and occupational health perspectives. These findings underline the necessity of strict implementation of dust control measures, appropriate personal protective equipment, and effective ventilation and extraction systems during e-waste crushing and subsequent separation processes.

## 4. Discussion

Metals in electronic waste occur not only as larger components such as heat sinks, metal sheets, wires, and large connectors, but also as numerous small elements including fine wires, pins, contacts, and solder joints. In printed circuit boards (PCBs), a wide range of metals is incorporated into the composite structure consisting of a polymer matrix reinforced with glass fibres and copper conductive layers [14,27].

During mechanical processing of WEEE, particularly crushing and milling, these heterogeneous structures undergo fragmentation, which leads to the liberation of small metallic particles from the surrounding non-metallic matrix. As a result of this comminution process, metallic particles are progressively reduced in size and released into finer particle fractions. Due to their higher density compared to polymeric and ceramic components, these liberated metal particles tend to concentrate in fine fractions, dust, and airborne particles generated during processing [13,16].

Fine particulate fractions therefore represent an important carrier of metals in mechanically processed e-waste streams. From the perspective of resource recovery, these dust fractions may represent a valuable secondary raw material within the concept of urban mining and circular resource utilization [12,27]. At the same time, however, they also represent a potential environmental and occupational health risk, as they often contain elevated concentrations of hazardous heavy metals such as lead, cadmium, nickel, and tin, as demonstrated in the present study.

The observed enrichment of metals in fine particle fractions can be attributed to the combined effects of mechanical crushing and the structural characteristics of electronic components. During size reduction, metallic phases associated with solders (Pb–Sn systems), conductive tracks (Cu, Ag), and thin surface coatings or contact layers (Au, Ag) are preferentially liberated from composite structures and polymeric substrates. These phases are typically brittle or weakly bonded, which promotes their fragmentation and subsequent accumulation in finer size fractions. As a result, fine fractions become enriched not only in base metals but also in hazardous and precious elements.

In addition to the preferential liberation of metallic phases, fine particles exhibit a significantly higher specific surface area compared to coarse fractions. This enhances the apparent concentration of metals associated with surface-bound phases, corrosion products, and thin metallic layers. Consequently, even a relatively small mass of fine material may contain a disproportionately high share of the total metal inventory of the processed e-waste.

Although the finest fractions represent only a minor proportion of the total mass of crushed e-waste, they are disproportionately enriched in hazardous metals and respirable particles. The coexistence of PM10 and PM2.5 particle size ranges with elevated contents of toxic elements such as Pb, Cd, As, Sn, Ni, and Cr represents a significant occupational and environmental risk. Particles within these size ranges can readily become airborne during crushing, sieving, and handling operations and may be inhaled by workers or dispersed into the surrounding environment. The SEM–EDS results confirm the presence of metal-bearing particles at micrometre and sub-10 µm scales, supporting the conclusion that hazardous metals are present in forms that are potentially bioavailable and mobile.

From an environmental perspective, fine particles enriched in heavy metals may act as carriers facilitating the transport of toxic substances over long distances once released into the environment. Moreover, some metals present in e-waste may undergo chemical or biological transformations that increase their toxicity, further amplifying their potential impact. These findings emphasize the necessity of strict implementation of dust control measures, effective ventilation systems, and appropriate personal protective equipment during e-waste processing.

At the same time, fine fractions exhibit a dual character. While they pose elevated health and environmental risks due to the concentration of hazardous elements, they are also enriched in economically valuable metals such as silver, gold, copper, and aluminium. This duality highlights the importance of controlled and well-designed processing strategies that simultaneously minimize occupational exposure and enable efficient recovery of valuable metals. Fine fractions should therefore not be regarded merely as an undesirable by-product of mechanical processing, but rather as a material stream requiring specialized handling and targeted treatment.

From a resource recovery perspective, fine-grained fractions enriched in metals represent a potentially valuable secondary raw material. Several technological approaches can be applied for the recovery of metals from such fractions. Physical separation techniques, including density separation, magnetic separation, and electrostatic separation, are commonly used as preliminary concentration steps to separate metal-rich particles from non-metallic materials. Subsequently, hydrometallurgical processes such as acid leaching, solvent extraction, and selective precipitation are frequently applied for the recovery of base and precious metals such as Cu, Ag, and Au. Alternatively, pyrometallurgical treatment routes involving smelting and refining processes can also be used to recover metals from complex e-waste streams. The selection of an appropriate processing route depends on factors such as particle size distribution, metal composition, and economic feasibility [7,10].

The size-dependent enrichment trends observed in this study are consistent with previously published findings on the processing and recycling of electronic waste. The combined application of sieve analysis, particle size distribution measurement, ED-XRF chemical analysis, and SEM–EDS elemental mapping provides a comprehensive insight into both the bulk composition and particle-scale distribution of metals in e-waste. This integrated approach allows for a more robust assessment of both risks and resource potential, underlining the critical importance of fine fraction management in modern e-waste recycling and circular economy strategies.

## 5. Conclusions

This study presents a detailed evaluation of the size-dependent distribution of heavy metals in mechanically processed e-waste fractions. The results demonstrate that the investigated e-waste contains a wide range of heavy and potentially hazardous metals whose concentrations are strongly dependent on particle size.

A pronounced enrichment of metals was observed in the finest fractions generated during crushing and sieving. Compared with the coarse fraction (>3 mm), Zn concentrations increased by approximately one order of magnitude, while Cr, Ni, and Cd exhibited increases of up to approximately twentyfold in the finest fractions. Pb showed the highest enrichment, reaching concentrations of approximately 2 wt.% in the <0.15 mm fraction, corresponding to several ten-fold enrichment relative to coarse fractions. Sn also showed substantial enrichment, in some cases increasing by up to two orders of magnitude. In addition, measurable concentrations of As and Se were detected in the finest fractions, whereas Hg was not detected in any analysed sample.

Particle size distribution analysis revealed that the fine dust generated during mechanical processing contains a significant proportion of respirable particles, including approximately 14–15 vol.% PM10 and about 3.5 vol.% PM2.5, confirming the potential for inhalation exposure during e-waste processing. Combined ED-XRF and SEM–EDS analyses further demonstrated that hazardous metals such as Pb and Sn occur in particles at micrometre and sub-10 µm scales.

The results provide a size-resolved characterization of heavy metal distribution in crushed e-waste fractions, demonstrating that although fine fractions represent only a minor mass proportion of the processed material, they act as the dominant carriers of hazardous metals and respirable particles generated during mechanical treatment. At the same time, these fractions are enriched in valuable metals such as Ag, Au, Cu, and Sn, highlighting their dual role as both a potential environmental risk and a secondary re-source. These findings underline the importance of controlled handling, effective dust management, and targeted processing strategies during e-waste recycling. Future research should focus on a more detailed characterization of metal bioavailability in respirable particles and on the development of optimized processing technologies enabling safe handling and efficient recovery of valuable metals from fine e-waste fractions.

## Figures and Tables

**Table 1:** Content of selected elements in separated fractions of e-waste.

Fraction (mm)	Content of Selected Elements (ppm/wt.%)														
	Al	Cu	Si	Cr	Mn	Fe	Ni	Zn	Sn	Sb	Pb	Cd	Tl	Ag	Au
1 (>3)	1.97	0.12	0.71	173	435	2161	67	1116	107	1977	368	18	5457	45	661
2 (3–1)	5.62	1.03	1.15	202	508	2368	86	1589	497	1763	1373	18	6973	44	758
3 (1–0.5)	8.21	3.63	2.10	325	677	3602	143	3371	2190	2456	3094	119	8165	136	844
4 (0.5–0.25)	11.77	10.90	3.95	315	882	5026	451	3458	4768	2076	6366	185	9347	365	823
5 (0.25–0.15)	10.04	10.23	5.86	960	2738	1.28	1073	8876	1.58	2243	2.07	811	5845	1377	548
6 (<0.15)	5.99	6.77	5.90	1747	8435	4.04	1600	1.48	2.79	2607	2.65	1415	4282	2314	429
Fine dust	4.43	2.02	6.46	1235	7369	3.14	965	7241	2847	345	6340	153	1769	280	161

Due to the wide range of element concentrations, values are reported either in ppm or wt.%. Values expressed in wt.% are highlighted in grey.

## Data Availability

The original contributions presented in this study are included in the article. Further inquiries can be directed to the corresponding author.

## Abbreviations

The following abbreviations are used in this manuscript:

ED-XRF	Energy Dispersive X-ray Fluorescence
SEM–EDS	Scanning Electron Microscopy–Energy Dispersive Spectroscopy
e-waste	Waste Electrical and Electronic Equipment
PM10	Particulate Matter 10 micrometers (µm) or less in diameter
PM2.5	Particulate Matter 2.5 micrometers (µm) or less in diameter
WEEE	Waste Electrical and Electronic Equipment
RoHS	Restriction of Hazardous Substances
OM	Optical microscopy
PSD	Particle size distribution
PCB	Printed circuit board
